# Using alcohol consumption diary data from an internet intervention for outcome and predictive modeling: a validation and machine learning study

**DOI:** 10.1186/s12874-020-00995-z

**Published:** 2020-05-11

**Authors:** Philip Lindner, Magnus Johansson, Mikael Gajecki, Anne H. Berman

**Affiliations:** 1 Centre for Psychiatry Research, Department of Clinical Neuroscience, Karolinska Institutet, & Stockholm Health Care Services, Stockholm County Council, Stockholm, Sweden; 2 Center for Dependency Disorders, Stockholm Health Care Services, Stockholm County Council, Stockholm, Sweden; 3 Department of Psychology, Stockholm University, Stockholm, Sweden; 4 Department of Public Health, Karolinska Institutet, Stockholm, Sweden; 5 Department of Psychology, Uppsala University, Uppsala, Sweden

**Keywords:** Alcohol, Measurement, Calendar, Diary, Machine learning, Classification, Prediction

## Abstract

**Background:**

Alcohol use disorder (AUD) is highly prevalent and presents a large treatment gap. Self-help internet interventions are an attractive approach to lowering thresholds for seeking help and disseminating evidence-based programs at scale. Internet interventions for AUD however suffer from high attrition and since continuous outcome measurements are uncommon, little is known about trajectories and processes. The current study investigates whether data from a non-mandatory alcohol consumption diary, common in internet interventions for AUD, approximates drinks reported at follow-up, and whether data from the first half of the intervention predict treatment success.

**Methods:**

*N* = 607 participants enrolled in a trial of online self-help for AUD, made an entry in the non-mandatory consumption diary (total of 9117 entries), and completed the follow-up assessment. Using multiple regression and a subset of calendar data overlapping with the follow-up, scaling factors were derived to account for missing entries per participant and week. Generalized estimating equations with an inverse time predictor were then used to calculate point-estimates of drinks per week at follow-up, the confidence intervals of which were compared to that from the measurement at follow-up. Next, calendar data form the first half of the intervention were retained and summary functions used to create 18 predictors for random forest machine learning models, the classification accuracies of which were ultimately estimated using nested cross-validation.

**Results:**

While the raw calendar data substantially underestimated drinks reported at follow-up, the confidence interval of the trajectory-derived point-estimate from the adjusted data overlapped with the confidence interval of drinks reported at follow-up. Machine learning models achieved prediction accuracies of 64% (predicting non-hazardous drinking) and 48% (predicting AUD severity decrease), in both cases with higher sensitivity than specificity.

**Conclusions:**

Data from a non-mandatory alcohol consumption diary, adjusted for missing entries, approximates follow-up data at a group level, suggesting that such data can be used to reveal trajectories and processes during treatment and possibly be used to impute missing follow-up data. At an individual level, however, calendar data from the first half of the intervention did not have high predictive accuracy, presumable due to a high rate of missing data and unclear missing mechanisms.

## Background

High consumption of alcohol, with and without symptoms of alcohol use disorder (AUD) [1], is a large public health issue and the third largest contributor to the global burden of disease [2]. Approximately one in ten adult males and one in twenty adult females, report drinking at harmful levels [3], and AUD has an estimated lifetime prevalence of nearly 30% [4]. Despite this, only around 15% will seek treatment [5]. Reasons for not doing so include denial of problems, a desire to overcome difficulties by oneself, antagonisms towards historically dominant treatment options, and shame [6–8].

Evidence-based internet interventions for hazardous drinking and AUD are an attractive way of meeting this clinical and public health challenge and can be delivered via online platforms or smartphone applications [9]. These interventions, often based on cognitive behavioral therapy (CBT) and/or motivational interviewing (MI) components, can be designed both as both open, low-intensity interventions with less structure and adherence requirements, typically without guidance from an online therapist; or as high-intensity interventions that are more structured and demanding, and almost always include regular feedback and support from an online therapist [10]. Historically, internet interventions for AUD have been dominated by the low-intensity format, typically resulting in low effect sizes [11], but with the advantage of having unlimited scalability; this is in contrast to the psychiatry field, where high-intensity formats are the norm and greater effect sizes are observed, comparable to face to face [12].

A common component of internet interventions for AUD is a digital alcohol consumption diary [13, 14], also called calendar, that users can use to record drinking as well as situational parameters over time, gaining insight not only into their total consumption of alcohol, but also behavior patterns associated with drinking (such as drinking alone, when in a depressed mood, etc.). These diaries are typically not mandatory, but in terms of data, provide information equivalent to the last-week timeline follow-back (TLFB) measurement [15] often used to derive outcome measures in clinical trials (e.g. total drinks per week or number of drinking days) [16]. Despite clear disadvantages, many trials still rely on a simple pre-post measurement strategy [17]. Collecting outcome data continuously throughout the intervention duration, e.g. weekly [18], allows for more advanced statistical modeling techniques capable of estimating different trajectories of change and estimating missing data appropriately [19]. The latter is especially important since internet interventions for AUD often suffer from high levels of attrition [20], making it preferable to collect outcome data during the intervention that can help either model or impute missing outcomes. Continuous outcome measurement would also enable investigations into the mechanisms of change in treatment [21].

Internet interventions that include an alcohol diary already collect data that can be used for continuous outcome modeling. However, since use of the diary is typically not mandatory, high rates of missing data are probable. Further, the missing data mechanism is not obvious and likely to differ across individuals and over time: while missing data for AUD or psychiatric symptoms is never assumed to equal zero symptoms, the equivalent may very well be the case for drinking (i.e. some participants not reporting drinking for lack thereof). For this reason, using data collected from a non-mandatory alcohol diary for outcome modeling needs to first be validated before being used for other analyses. If the diary data is indeed found to suitable for outcome modeling, it may also have predictive value. Previous research on predicting outcomes in behavioral treatments for AUD have relied on baseline data, and model accuracies are seldom much above chance [22]. Predicting outcomes based on data from the first half of the intervention is an attractive alternative and would have greater clinical value. In particular, accurate predictions could serve as a cost-effective decision support tool to inform adjustments to intervention content and format in order to avoid undesired outcomes. An obvious candidate for adjustment is availability and degree of therapist support. Therapist support in internet interventions for AUD is typically associated with somewhat greater effect sizes [23], although the exact causal mechanisms through which the effect is mediated remains unknown [10]. Since internet interventions for AUD typically do not feature therapist support, providing participants with predicted poor outcomes with therapist support may be a cost-effective way of tailoring intervention delivery to individual needs. In interventions where some degree of therapist support is provided to all participants, those with predicted poor outcomes may be offered more or a different type of support. A recent randomized trial on insomnia provides proof-of-concept that individual predictions and resulting adaptations to the internet intervention can be used to avoid undesired outcomes [24].

In the current study, we first used consumption calendar data from a large (*n* = 4165) low-intensity internet intervention for AUD [25] to evaluate whether the trajectory-derived point-estimate approximated the follow-up assessment. Second, we examined the classification accuracies of random forest machine learning in predicting treatment success from summary measures of calendar data collected during the first half of the intervention. Random forest classification was chosen since it can incorporate many types of features, is robust to outliers (through binarization) and provides intelligible importance ratings [26].

## Methods

### Participants

Participants in the current study were included in a trial on online self-help for AUD [25]. Trial participants were recruited during a period of two years, had to score ≥ 6/8 (women and men, respectively) on the Alcohol Use Disorder Identification Test (AUDIT) [27] to be included, and were given access to a self-paced, eight-module self-help program [28] based on cognitive behavioral therapy, with motivational interviewing components [29]. Program users were informed of consumption diary and encouraged to use it, but were not actively prompted to do so. Out of the *n* = 4165 participants who began the trial, *n* = 1043 completed the follow-up assessment (distributed ten weeks post-baseline or three weeks after completing the last module), and where eligible for inclusion in the current study. Sample characteristics are provided in the trial report [25]. Out of the *n* = 1043 who completed the follow-up, *n* = 607 used the calendar at least once during the intervention period, providing k = 9117 diary entries after removal of duplicates (maximum reported drinks per day and user retained) and entries outside the intervention duration.

### Measures and data preprocessing

At baseline and the follow-up, participants recorded last-week consumption of standard drinks of alcohol using the TLFB method. While it is possible to derive a variety of metrics from the TLFB measure, we focused exclusively on total number of (standard) drinks since this is a common outcome in trials [16] and appears prominently in national guidelines [30]. More importantly, one could argue that the total number of drinks metric is more robust to missing data than e.g. number of heavy drinking days, since there are fewer missingness assumption (participant either drank zero or more on missing day, as opposed to participant either drank zero or more than X). Equivalent data on last-week total number of drinks were compiled from calendar data by calculating number of days into treatment for each entry (based on baseline and follow-up dates), and collapsing the data using a summary function into weeks, while saving number of days included per week (1–7, or missing week) as a separate variable for each individual and week.

**Fig. 1:**
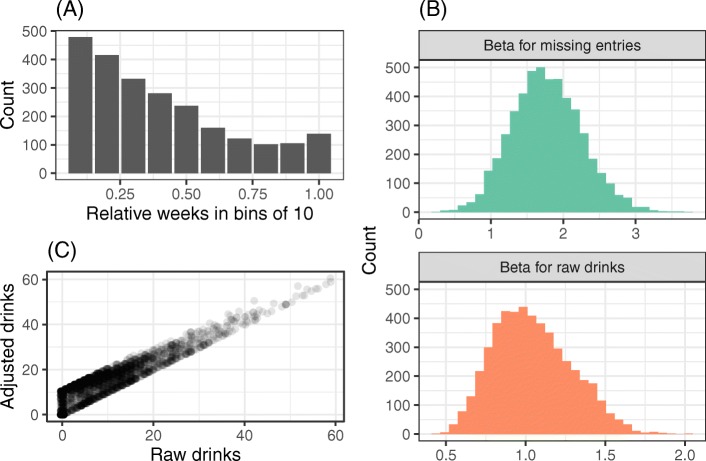
Data preprocessing. (**a**) Counts of entries per 10-bins of normalized intervention duration. (**b**) Bootstrapped distributions of parameter estimates used to adjust raw drinks. (**c**) Scatterplot of adjusted drinks and raw drinks, with alpha set to 0.1 and random jitter added to display overlap

A total of k = 2372 participant weeks were compiled, with an average of M = 3.91 (SD = 2.80) weeks per participant, ranging from one to 16 weeks included. See Fig. 1 panel A for distributions (binned into quantiles of 10 normalized by intervention duration). To evaluate whether it was possible to adjust for missing entries in the calendar, and evaluate the appropriateness of the missing at random mechanism, an adjustment algorithm was derived using multiple regression and a subset of participants (*n* = 92, k = 259 underlying entries) who provided calendar data that overlapped with the TLFB period at follow-up. As expected, this subset of participants had a significantly higher number weeks of data (M = 6.84 vs M = 3.38, d = − 1.37 [− 1.61—-1.14]) and underlying entries (M = 31.6 vs M = 12.1, d = 1.28 [− 1.52—-1.05]). Importantly however, between-group differences in average drinks (M = 8.21 vs M = 8.97, d = 0.11 [− 0.12—0.33]) and maximum drinks per week (M = 13.3 and M = 16.2, d = − 0.26 [− 0.49—-0.04] were small in magnitude, suggesting no meaningful sampling bias.

This single-imputation approach with empirically-derived adjustment factors was chosen over alternatives like multiple imputation due to the complexity of the data – varying, likely non-random number of entries with known structural properties (number of missing entries per week) – and high percentage of missing data [31]. The utilized regression model predicted TLFB-reported drinking from calendar-reported drinking and number of days missing from the calendar week: the resulting beta coefficients (excluding a negative, non-significant intercept) were used to calculate adjusted total drinks for each entry in the calendar, under the assumption that the association between calendar-reported drinking and TLFB-reported drinking is the same throughout the intervention period. The appropriateness of this assumption is tested by comparing the trajectory-derived point-estimate with the follow-up assessment. Stability of the beta coefficients was examined using bootstrapping (5000 repeats; see Fig. 1 panel B for distributions) and inspecting the scatterplot of predicated and observed values at follow-up for outliers (see Fig. 1 panel C).

### Statistical analyses

All analyses were conducted in the R (3.6.1) statistical environment; complete R code is available at 10.17605/OSF.IO/FDVM7. For the validation part of the study, we first plotted the summarized weekly data to inspect non-linear trends. Second, we used gaussian-family generalized estimating equations (GEE) with robust Sandwich estimators and an exchangeable correlation structure, as implemented in the *geepack* R package [32], to estimate population-average trends in drinking (both raw and adjusted) over a standardized treatment duration (a numeric time predictor), calculated as week X / total weeks (to account for differences in when participants completed the follow-up assessment). The TLFB-derived mean at the follow-up assessment was calculated and compared to the GEE-predicted point-estimates (raw and adjusted). Good estimation was defined as an overlap between the 95% confidence interval of the GEE-predicted point-estimate with the confidence interval of the TLFB-derived mean; otherwise, an over- or under-estimation would be present. Of note, paired measurements with overlapping confidence intervals of the individual means may still differ significantly from each other depending on the distribution of difference scores (not available in our study since the point-estimate from the GEE model is predicted on a group-level using an inverse time variable, and not observed on an individual level).

For the second part of the study, where we explored whether the calendar data had predictive value, we retained calendar data from the first half of treatment and calculated a variety of summary and difference measures, both absolute and relative to number of entries (e.g. percentage of entries considered heavy drinking). Two additional features (random slope and intercept) came from a Poisson mixed model [33] with a linear time trend, in order to capture reliable individual trajectories, totaling *n* = 18 features. See Table 1 for descriptive statistics on included predictors. As in the original trial [25], two binary classification targets were examined in separate models: non-hazardous drinking at follow-up (≤14 standard drinks for men and ≤ 9 for women) and change in AUDIT severity group [27]. Out of the *n* = 582 participants with any data during the first half of the intervention (<.5 of normalized intervention duration), *n* = 59 participants were excluded due to taking more than 35 extra days (after the intended 70) to complete the follow-up assessment (final k = 5735). Machine learning was performed in two steps. First, using the *caret* R package [34], two random forest machine learning models (one for each outcome, both with repeated cross-validation [RCV]) were trained on the full dataset (predictors scaled and centered) with the standard *n* = 500 trees and systematic grid search (1—17) for evaluation of optimal number of variables randomly sampled at each split, as determined by ROC values.. Specificity and sensitivity metrics, calibration curves and scaled variable importance metrics (0–100) were extracted from the optimal models. Second, potential generalizability was examined using nested cross-validation (NCV) [35] (10 outer folds, 10 inner folds) with the default AUC cutoff of 0.5.

**Table 1:** Summary variables used to train machine learning algorithms

Variable name	Description	Mean	Median	Max	Min	SD
Abs.diff	Absolute difference first-last reported drink	−0.39	0	12	−17	3.14
Avg.drinks	Average reported drinks	3.26	3	15	0	2.42
Entries	Total number of entries	10.97	8	48	1	9.73
intercept	Intercept of trajectory	0.05	0.22	4.79	−2.73	1.13
IQR.drinks	Inter-quartile range of drinks	1.69	1	13.5	0	1.89
Max.drinks	Maximum reported drinks	6.05	6	22	0	3.98
Median.drinks	Median reported drinks	2.92	3	15	0	2.62
Min.drinks	Minimum reported drinks	1.67	1	15	0	2.24
n.binge	Number of binge drinking entries	0.72	0	20	0	1.71
n.heavy	Number of heavy drinking entries	2.35	1	35	0	3.32
n.light	Number of light drinking entries	7.9	4	48	0	8.79
Perc.binge	Percentage binge drinking entries	0.09	0	1	0	0.2
Perc.heavy	Percentage of heavy drinking entries	0.26	0.17	1	0	0.3
Perc.light	Percentage of light drinking entries	0.65	0.75	1	0	0.35
Range.drinks	Range of reported drinks	4.39	4	22	0	4.15
Rel.diff	Relative difference first-last reported drinks	−0.06	0	2.5	−4.5	0.61
slope	Slope of trajectory	−0.01	−0.01	9.21	−14	2.34
Sum.drinks	Total sum of reported drinks	28.29	16	208	0	32.23

^1^Bing-drinking defined as > 6 for women, > 8 for men

^2^Heavy drinking defined as > 3 and < 7 for women, > 4 and < 9 for men

^3^Light drinking defined as < 4 drinks for women, < 5 for men

## Results

### Validation

**Fig. 2:**
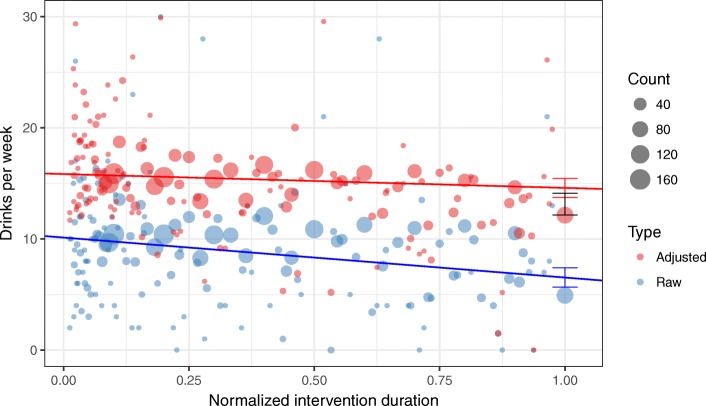
Raw and adjusted point-estimates from calendar data vis-a-vi follow-up. Y-axis cropped at 30 drinks per week for visualization purposes (*n* = 8 points omitted)

The beta coefficients used to adjust the raw data were calculated to 1.73 drinks per missing day per week and 0.996 predicted drinks per reported drink. GEE modeling of the raw calendar data revealed a significant effect of time corresponding to a reduction of 3.60 standard drinks of alcohol (SE = 0.61, *p* < .001), with an end-point estimate of B = 6.53 (SE = 0.45, p < .001). GEE modeling of the adjusted calendar data revealed a significant effect of time corresponding to a reduction of 1.25 drinks of alcohol (SE = 0.62, *p* = .044), with an end-point estimate of 14.59 (SE = 0.44, *p* < .001). The adjusted end-point estimate, but not the raw end-point estimate, overlapped with the TLFB-derived point-estimate (B = 13.13, SE = 0.50). See Fig. 2.

### Predictive accuracy

**Fig. 3:**
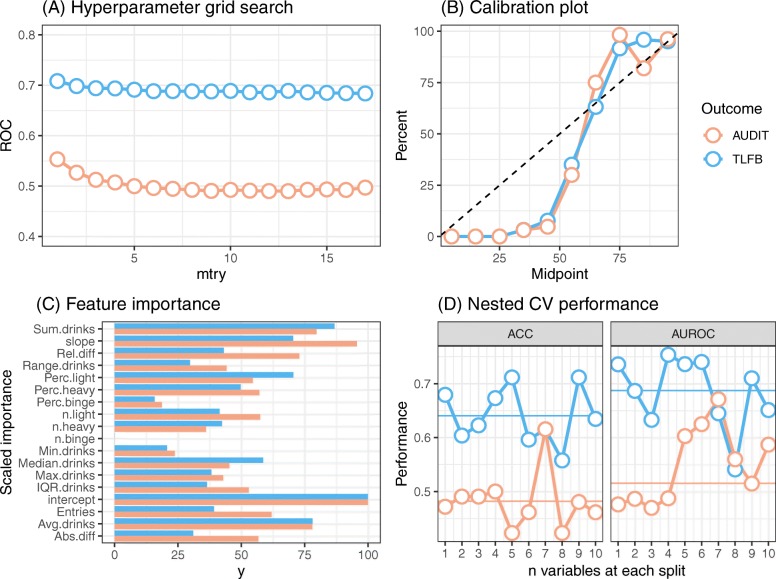
Results of machine learning models

Agreement between improvement in non-hazardous drinking and AUDIT severity was 62%. Average NCV model accuracy was higher in predicting non-hazardous drinking (64% accuracy) than AUDIT severity improvement (48% accuracy), and in both cases, sensitivity was markedly higher than specificity, especially for non-hazardous drinking improvement. See Table 2. In both prediction models, random slope and intercept, along with sum and mean of reported drinks had the highest model importance. The largest absolute between-outcome difference in importance were found in first-last drink absolute and relative differences, slope, and number of entries. See Fig. 3.

**Table 2:** Machine learning prediction accuracies

Variable	Predicting non-hazardous consumption (***n*** = 290, 55.4%)	Predicting lowered AUDIT severity group (***n*** = 269, 51.4%)
NCV average accuracy	0.64	0.48
NCV average ROC	0.69	0.52
RCV sensitivity	0.79	0.63
RCV specificity	0.53	0.44

*NCV* Nested cross-validation models. *RCV* repeated cross-validation. *ROC* Receiver operating characteristics

## Discussion

The current study sought to first validate the use of data from a non-mandatory alcohol consumption diary, collected during an internet intervention, for outcome modeling, with the follow-up assessment used as gold standard. Results showed that while the raw calendar data substantially underestimated sample-average drinking at follow-up, adjusting the raw data using an empirically-derived scaling factor from overlapping data – accounting for both reported drinking and missing data – resulted in calendar-reported sample-average drinking that was similar to that reported at the follow-up. Having shown that the adjusted calendar data approximated follow-up outcome data, we then tested whether calendar data from the first half of the intervention could be used to predict successful outcomes at follow-up, an insight that in future research could be used to adapt the remaining intervention in order to reduce the likelihood of unsuccessful outcomes [24]. The two random forest machine learning models achieved classification accuracies of 64 and 48%, in predicting improvement in non-hazardous drinking and AUDIT severity, respectively. In all, this suggests that while data from a non-mandatory alcohol consumption calendar does approximate formal outcome assessment at a group level (after adjustment), using this data to predict outcomes on an individual level led did not lead to high accuracies.

Assuming that the empirically derived scaling factors are equally applicable throughout the intervention duration (as suggested by the point-estimate approximating the follow-up assessment), our analyses reveals a sharp immediate decrease in drinking after intervention enrollment and a small linear decrease after that. In the original trial, those later lost to follow-up reported an average of 28.1 (SD = 18.66) drinks per week on the baseline TLFB. Our findings suggest that by week one, the sample is down to approximately 16 drinks per week, which is unlikely to be an effect of the intervention itself but rather an effect of treatment-seeking occurring within a motivational window opened by negative consequences, as is common in the addiction field [36]. Importantly, treatment could still have an effect in such a case by keeping participants motivated and by teaching behavioral strategies to avoid relapse. Finding a steep decrease after intervention enrollment is not uncommon in the addiction field (e.g. [37]), but our findings do deviate from research on a high-intensity version of the same online treatment program for AUD, that showed a linear but fluctuating decrease from screening and forward [18]. The high-intensity format attracting a different type of participant, and/or lower rates of missing data and non-random missingness in formal continuous outcome measurements, could explain this discrepancy.

Prima facie, finding a steep decrease early in treatment would appear to suggest good accuracy of the initial trajectory in predicting final outcomes. However, we found classification accuracies only moderately above chance and roughly equal to the accuracies found in the original study (66 and 60%) that used logistic regression model and only baseline data [25]. Finding higher accuracy for the TLFB-related than AUDIT severity-related classification is to be expected since the underlying data is the same; the calendar did not allow reporting of negative consequences per drinking occasion. Relatively low prediction accuracies are likely explained by a high degree of missing data (85.5%), the missing mechanism of which is likely to differ between individuals and over time. Higher sensitivity than specificity in both prediction models indicate that many people start off well (in motivational window) but later relapse. Predicting treatment outcomes in addiction has historically proven challenging [22], and while the growing ubiquity of machine learning promises to improve prediction accuracies [38], a recent systematic review found that clinical prediction models typically do not perform better than logistic regression [39].

### Limitations

Several limitations apply to the analyses described herein. First, since analyses relied on data from the follow-up assessment as gold standard, *n* = 2854 participants enrolled in the trial were excluded. Importantly, as described in the original trial, this missingness cannot be considered missing at random since several differences in baseline characteristics were observed between participants that did and did not complete the follow-up [25]. However, to what extent this missingness pattern restricts the applicability of the derived scaling algorithm remains to be evaluated. Second, applicability of the empirically derived scaling factors throughout the intervention duration can only be inferred from the overlap with the follow-up measure at the endpoint, but not tested directly. Third, a limited set of predictors, a relatively small sample and only one type of machine learning model was evaluated, although it should be noted that evaluating the optimal machine learning model for this particular type of data and situation was not part of the research question.

## Conclusions

Data from a non-mandatory alcohol consumption diary, adjusted for missing entries, approximates follow-up data at a group level, suggesting that such data can be used to reveal trajectories and processes during treatment and possibly be used to impute missing follow-up data. At an individual level, however, calendar data from the first half of the intervention did not have high predictive accuracy, presumable due to a high rate of missing data and unclear missing mechanisms.

## Data Availability

The datasets used and/or analyzed during the current study are available from the corresponding author on reasonable request.

## Abbreviations

AUC: Area Under the Curve
AUD: Alcohol Use Disorder
AUDIT: Alcohol Use Disorder Identification Test
GEE: Generalized Estimating Eqs.
NCV: Nested Cross-Validation
RCV: Repeated Cross-Validation
ROC: Receiver operating characteristics
TLFB: Timeline Follow-Back
